# Synthesis of a novel multifunctional organic–inorganic nanocomposite for metal ions and organic dye removals

**DOI:** 10.1038/s41598-023-38420-2

**Published:** 2023-08-08

**Authors:** Ahmed Elmekawy, Qui Quach, Tarek M. Abdel-Fattah

**Affiliations:** 1 Applied Research Center at Thomas Jefferson National Accelerator Facility and Department of Molecular Biology and Chemistry at Christopher, Newport University, Newport News, VA 23606 USA; 2 Department of Physics, Tanta University, Tanta, Al Gharbiyah Egypt; 3 Faculty of Sciences, Alexandria University, P.O. Box 426, Ibrahimia, 21321 Alexandria Egypt

**Keywords:** Engineering, Nanoscience and technology, Chemistry, Environmental chemistry

## Abstract

In this study, we used solvent assisted mechano-synthesis strategies to form multifunctional organic–inorganic nanocomposites capable of removing both organic and inorganic contaminants. A zeolite X (Ze) and activated carbon (AC) composite was synthesized via state-of-the-art mechanical mixing in the presence of few drops of water to form Ze/AC. The second composite (Ze/L/AC) was synthesized in a similar fashion, however this composite had the addition of disodium terephthalate as a linker. Both materials, Ze/AC and Ze/L/AC, were characterized using scanning electron microscope (SEM), energy-dispersive X-ray spectroscopy (EDS), Powdered X-ray diffraction (P-XRD), Fourier-transform infrared spectrometry (FTIR), Accelerated Surface Area and Porosimetry System (ASAP), and thermal gravimetric analysis (TGA). The SEM–EDS displayed the surface structure and composition of each material. The sodium, oxygen and carbon contents increased after linker connected Ze and AC. The P-XRD confirmed the crystallinity of each material as well as the composites, while FTIR indicated the function groups (C=C, O–H) in Ze/L/AC. The contaminant adsorption experiments investigated the effects of pH, temperature, and ionic strength on the adsorption of methylene blue (MB) and Co(II) for each material. In MB adsorption, the first-order reaction rate of Ze/L/AC (0.02 h^−1^) was double that of Ze/AC (0.01 h^−1^). The reaction rate of Ze/L/AC (4.8 h^−1^) was also extraordinarily higher than that of Ze/AC (0.6 h^−1^) in the adsorption of Co(II). Ze/L/AC composite achieved a maximum adsorption capacity of 44.8 mg/g for MB and 66.6 mg/g for Co(II) ions. The MB adsorption of Ze/AC and Ze/L/AC was best fit in Freundlich model with R^2^ of 0.96 and 0.97, respectively, which indicated the multilayer adsorption. In the Co(II) adsorption, the data was highly fit in Langmuir model with R^2^ of 0.94 and 0.92 which indicated the monolayer adsorption. These results indicated both materials exhibited chemisorption. The activation energy of Ze/L/AC in MB adsorption (34.9 kJ mol^−1^) was higher than that of Ze/L/AC in Co (II) adsorption (26 kJ mol^−1^).

## Introduction

Pollution caused by the excessive use of heavy metal ions and organic matter in industrial processes has directly and indirectly led to the discharge of waste materials into waterways, contaminating ecosystems and affecting human life^1–3^. Heavy metal ions like cobalt (II), nickel (II), chromium (VI), lead (II), and others can be toxic to life even in low concentrations^4–7^. The toxicity of cobalt, nickel, chromium, and lead can occur at 7 µg/L, 100 µg/L, 50 µg/L, 5 µg/dL respectively^8,9^. Heavy metal like Cobalt is released into the environment in multiple ways including the use of non-radioactive Co in industrial processes and radionuclides ^60^Co and ^58^Co in medicine^5^. It was reported that the cobalt level in blood was associated to the increased muscle spasticity^8^. Similarly, high levels of organic pollutants such as dyes and phenolic components can be highly toxic. For instance, the intake of methylene blue (MB) can cause profuse sweating, nausea, vomiting, neuronal apoptosis, burning sensations and many other negative health effects^10,11^.

There have been various effective methods applied to filter heavy metals and dyes including reverse osmosis, distillation, and adsorption. However, it is difficult to find a cheap and effective method. For example, reverse osmosis is the most common method applied in filtering water, but the method often wastes a significant amount of water^12^. Furthermore, the reverse osmosis process always removes a large amount of healthy minerals in water^12^. Distillation is another common method, but it can only filter small amounts of water and cost more energy^13^. Among the methods, adsorption is still the most effective method due to its high efficiency and being inexpensive^13^. The adsorbents can be cheaply produced from low-cost materials like agricultural waste, nanomaterials, and polymers^13^. These adsorbents can be physically and chemically engineered to filter specific contaminants. For example, various types of metal organic framework (MOF) materials including Zr-MOFs, ZIF-8, ZIF-67, and KIUB-MOF-1 have been designed to remove specific metals such as lead, mercury, methylene blue, and methyl orange^14–16^.

Among the adsorbents, Carbon based materials and silica materials have been widely utilized in various environmental remediation due to their non-toxic nature and low cost^17–19^. One such silica-based material, known as zeolite, is an inorganic material consisting of aluminum-silicates which share oxygen atoms, forming uniform cages, cavities, or channels and can be used as an effective absorbent for removing of heavy metals^19–27^. Many studies have shown that carbon-based materials like activated carbon can be used as an effective absorbent for the removal of organic materials such as methylene blue in a high capacity^31,32^. The large surface area of carbon-based materials also support their ability in capturing heavy metals^33,34^. Recently, several studies have indicated that zeolite and activated carbon made from mechano-synthesis can be used in environmental applications^35–37^.

Mechanochemical synthesis are a type of green chemistry approach that typically require significantly less solvent usage (or sometimes none) compared to traditional syntheses from solution^35–38^. It is known in the mechanochemistry field that adding small quantities of a solvent to the solids ground, described in the literature as liquid-assisted grinding (LAG), can yield different products, as well as increase the mechanochemical reaction rates^35–38^. This process can offer alternative synthetic routes, occasionally yielding products not obtainable from solution chemistry^35–38^.

In this study, we synthesized a novel composite material via LAG mechano-synthesis by linking zeolites with activated carbon via disodium terephthalate to form a multifunctional material. We compared the adsorption performance of zeolite-activated carbon (Ze/AC) composite with zeolite-linker-activated carbon (Ze/L/AC). Both materials were tested for their adsorption capacities against MB and Co(II) ions under various temperature, pH solutions, time and in the presence of interference ions.

## Material and method

### List of materials

The following materials were purchased: Activated carbon (Calgon Carbon Corporation), Zeolite 13X (8–12 mesh, Aldrich Chemical Company), Disodium terephthalate (99%, Alfa Aesar), methylene blue (MB) powder (J. T Baker Chemical Company), and Cobalt (II) nitrate hexahydrate as source for Co(II) ions (99%, Acros Organics B.V.B.A.). All other chemicals are of analytical grade and were obtained from Aldrich Chemical Company (St. Louise, MO 68178, US).

### Preparation of the composites

#### Preparation of Ze/AC mixture via liquid-assisted grinding (LAG)

The zeolite X (Ze) (2.000 g) was grinded with 2.000 g of activated carbon (AC) and a few drops (0.5 ml) of Deionized water (DI, 18MΩ) for 30 min via an agate mortar and pestle. The resulting composite (Ze/AC) was then heated for 24 h at 100 °C.

#### Preparation of linked composites LAG

The synthesis of the Ze/L/AC composite was like the Ze/AC composite, however, in this synthesis 2.0 g disodium terephthalate (C_8_H_4_Na_2_O_4_) was grinded together with 2.0 g Ze and 2.0 g AC and then mixed with a few drops of water (0.5 ml). The product (Ze/L/AC) was dried in the oven at 100 °C for 24 h. The mass of the linker was doubled to synthesize the Ze/2L/AC product and dried at 100 °C.

### Characterization

The crystal structure of each material was examined by using powdered X-ray diffraction (P-XRD, Rigaku Miniflex II, Cu Kα X-ray, nickel filter, Tokyo, Japan), with a scanning range from 5° to 90°. The functional groups of each material were determined by Fourier transform infrared spectroscopy (FTIR, Shimadzu IR-Tracer 100, Kyoto, Japan). The thermal stability and decomposition of the composites were measured by thermogravimetric analysis (TGA, NETZSCH TG 209 F3). The surface areas and pore sizes of Ze/AC and Ze/L/AC were measured at 77 K by an Accelerated Surface Area and Porosimetry System (Micromeritics -ASAP 2020).

The concentrations of all solutions in the studies were determined by Ultraviolet–visible Spectrophotometer (UV–VIS, Vernier, Oregon, United States) at 665 nm for MB and 510 nm for Co(II) ions. Various known concentrations of MB (2 ppm, 4 ppm, 6 ppm, 8 ppm,10 ppm) and Co(II) (100 ppm, 250 ppm, 500 ppm, 750 ppm, 1000 ppm) were measured by UV–VIS. The results were applied in building the calibration curves for determining unknown concentrations.

The surface of each composite was scanned by using scanning electron microscopy (SEM, JEOL JSM-6060LV). During the scanning process, the weight percentage of each element in the composite was determined by energy-dispersive X-ray spectroscopy (EDS, Thermo Scientific UltraDry).

### Adsorbent screening study

For MB adsorption, 0.02 ± 0.001 g of each type of adsorbent (AC, Ze, Ze/AC, Ze/L/AC, Ze/2L/AC) was placed in 100 ml Nalgene bottles with 100.00 ml of 10 ppm MB solution. For Co(II) adsorption, 0.1 ± 0.001 g of each type of adsorbent (AC, Ze, Ze/AC, Ze/L/AC, Ze/2L/AC) was placed in 100 ml Nalgene bottles with 10.00 ml of 1000 ppm Co(II) solution. The bottles were placed on a reciprocating shaker at 125 rpm for 24 h at constant temperature (ambient room temperature, 294 K). Each trial was repeated three times. The adsorption capacity of each adsorbent was calculated by using Eq. (1) ^39^:

1 $$q_{e} = \left( {\frac{{C_{o} - C_{e} }}{m} } \right)V$$

where qe is the adsorption capacity of the adsorbent (mg/g), C_o_ is the initial concentration of MB or Co(II) in the solution (mg/L), C_e_ is final concentration of MB or Co (II) in solution (mg/L), V is the volume of the solution (L), m is the mass of the adsorbent (g).

### Isotherm study

In the MB isotherm study, various masses of Ze/AC and Ze/L/AC composites (0.04 ± 0.001 g, 0.06 ± 0.001 g, 0.08 ± 0.001 g, 0.1 ± 0.001 g) were mixed with 100 ml of 10 ppm MB solution in 100 ml Nalgene bottles. The bottles were shaken at 125 rpm for 24 h at 294 K. The Co(II) isotherm study was performed using different weights of Ze/AC and Ze/L/AC composite adsorbents (0.100 ± 0.001 g, 0.1500 ± 0.001 g, or 0.2000 ± 0.001 g, 0.300 ± 0.001 g) to which a constant volume (10.00 ml) of solution (1000 mg l^−1^ Cobalt), was applied in 100 ml Nalgene bottles. Bottles were then agitated at 125 rpm for 24 h at ambient conditions. Each trial was repeated three times.

The percent removal (R%) was calculated by using the Eq. (2) ^39^:

2 $$R{\%} = \left( {\frac{{C_{o} - C_{e} }}{{C_{o} }}{ }} \right){*}100$$

where R% is the percentage of MB or Co(II) removed by the adsorbent, C_o_ is the initial concentration of MB or Co(II) in the solution (mg/L), Ce is final concentration of MB or Co (II) in solution (mg/L), The Langmuir and Freundlich models were applied to compare and evaluate the adsorption isotherm of Ze/AC and Ze/L/AC. The statistical significance of data was evaluated by coefficient of determination (R^2^).

The Langmuir adsorption theory describes the adsorption process, where the adsorbate adsorption is limited to one molecular layer^31^. The linearized model equation is depicted in Eq. (3):

3 $$\frac{{{\text{C}}_{{\text{e}}} }}{{{\text{q}}_{{\text{e}}} }} = \frac{1}{{{\text{Q}}_{{\text{m}}} {\text{K}}_{{\text{L}}} }} + \left( {\frac{1}{{{\text{Q}}_{{\text{m}}} }}} \right){\text{C}}_{{\text{e}}}$$

where q_e_ is the adsorption capacity of the adsorbent (mg/g) which was calculated by Eq. (1), C_e_ is the equilibrium concentration of adsorbate (mg/L), Q_m_ is the saturated adsorptive capacity (mg/g), Kis the Langmuir constant (L/mg).

The Freundlich Isotherm model takes into consideration heterogeneous adsorption in which the active sites of the adsorbent surface are not energetically uniform^39^. The Freundlich linear Eq. (4) is shown below:

4 $${\text{lnq}}_{{\text{e}}} = {\text{ln}}K_{f} + \left( {\frac{1}{{\text{n}}}} \right){\text{lnC}}_{{\text{e}}}$$

where q_e_ is the sorption capacity at equilibrium (mg/g). C_e_ is the concentration of MB or Co (II) ions at equilibrium (mg/L). K_f_ (L/mg) and n are the Freundlich isotherm constants.

### Kinetic study

In the kinetic study, 0.02 ± 0.001 g of each composite (Ze/AC, Ze/L/AC) was mixed with 100 ml of 10 ppm MB solution in 100 ml Nalgene bottles. Bottles were agitated at 125 rpm at ambient conditions. The MB solutions were extracted and analyzed within 24 h. A total of 10–11 data points was collected for each composite within 24 h. Each trial was repeated three times.

T Co(II) kinetic study was performed by mixing 10 ml Co(II) solution of an initial concentration of 1000 mg l^−1^ with 0.1 ± 0.001 g of adsorbent in 100 ml Nalgene bottle. Bottles were agitated at 125 rpm at ambient conditions. Co(II) solutions were withdrawn and analyze within 24 h. A total of 10–11 data points was collected for each composite within 24 h. Each trial was repeated three times.

The pseudo-first-order and pseudo-second-order model were applied to evaluate the adsorption kinetics data of both composites. The statistical significance of data was evaluated by coefficient of determination (R^2^).

The pseudo-first-order equation is generally expressed as follows in Eq. (5) ^40^:

5 $$\ln \left( {q_{e} - q_{t} } \right) = lnq_{e} - k_{1} t$$

where q_t_ is the adsorption capacity at a given time t [h] (mg/g), q_e_ [mg/g] is the adsorption capacity at equilibrium (mg/g), k_1_ [h^−1^] is the first-order reaction rate constant.

The integrated rate law models how reactant and product concentrations vary with time. The linearized integrated rate law for a Pseudo first-order reaction Eq. (6) is shown:

6 $${\text{Ln}}\left[ {{\text{C}}_{{\text{t}}} } \right] = - k_{1} {*}t + {\text{Ln}}\left[ {{\text{C}}_{0} } \right]$$

where [C]_t_ = concentration of C at any time t, [C]_o_ = original concentration at initial time, k_1_ = [h^−1^] is the first-order rate constant of the adsorption process.

The pseudo-second-order equation is represented as follows in Eation (12)^40^:

7 $$\frac{1}{{[{\text{C}}_{{\text{t}}} ]}} = \frac{1}{{[{\text{C}}_{0} ]}} + {\text{k}}_{2} {\text{t}}$$

where [C]_t_ = concentration of C at any time t, [C]_o_ = original concentration at initial time, k_2_ = [mg^−1^⋅h^−1^] is the second-order rate constant of the adsorption process.

### pH study

For pH study, the pH of MB and Co(II) solutions were adjusted by dropwise addition of either 0.1 M NaOH or HCl and tested via a pH meter (Thermo Fisher Scientific, Orion 3 Star, Massachusetts, United States). 0.02 ± 0.001 g of each adsorbent (Ze/AC, Ze/L/AC) was added to 100.00 ml of 10 ppm MB solutions of various pH values (2, 4, 6, 8 and 10) in the Nalgene bottles. The bottles were then shaken at 125 rpm for 24 h at constant temperature (294 K). Each trial was repeated three times.

For the Co(II) study, 0.1 ± 0.001 g of each adsorbent Ze/AC mixture and Ze/L/AC composite introduced to 10.00 ml 1000 ppm Co(II) solutions at various pH values (2, 4, 6, 8, and 10). Each bottle was also shaken for 24 h at 125 rpm and at ambient conditions. Each trial was repeated three times.

### Ion competition study

In the ionic strength study conducted at various concentrations of KNO_3_ (0.1 M and 0.01 M) for MB and Co(II) solutions. For MB, 0.02 ± 0.001 g of each adsorbent was mixed with 100.00 mL of 10 mgl^−1^. For Co (II), 0.10 ± 0.001 g of each adsorbent was added to 10.00 mL of 1000 mgl^−1^ Co(II) solution. The bottles that contained the treated MB and Co(II) solution were shaken at 125 rpm overnight at ambient conditions. Each trial was repeated three times.

### Adsorption thermodynamics

The temperature effect study was conducted at 294 K, 303 K, 308 K and 313 K. For MB, 100.00 ml of 10 mgl^−1^ MB treated with 0.02 ± 0.001 g of each adsorbent in a temperature controlled shaking water bath for 24 h at speed of 5 rpm. For Co(II), 10.00 ml of 1000 mgl^−1^ Co(II) treated with 0.10 ± 0.001 g with each adsorbent at speed of 5 rpm. Each trial was repeated three times.

Equations 8, 9 and 10 were used to calculate thermodynamic parameters^41^. The statistical significance of data was evaluated by coefficient of determination (R^2^).

Equation 8 was used to calculate the fraction adsorbed:

8 $$K_{d} = \frac{F}{1 - F}$$

where F is the fraction of metal ions adsorbed at equilibrium in which F = (F_i_ − F_e_)/F_i_, F_i_ is initial fraction; F_e_ is fraction at equilibrium.

Gibbs free energy change (ΔG) (KJ/mol^−1^) is the thermodynamic parameter calculated by using Eq. (9) ^42^:

9 $${\Delta G} = - RTLnK_{d}$$

where ΔG is Gibbs free energy change (KJ/mol^−1^), K_d_ is the thermodynamic Langmuir constant for the adsorption process (L/mg), R is the universal gas constant (0.0083144 kJ.mol^-^LK^−1^),

Equation 10 was used to calculate, the entropy (ΔS) (kJ mol^−1^ K^−1^) and enthalpy (ΔH) (kJ mol^−1^)^42^:

10 $$lnK_{d} = \frac{{{\Delta S}}}{R} - \frac{{{\Delta H}}}{RT}$$

where ΔS is the entropy change (kJ mol^−1^ K^−1^), ΔH is the enthalpy change (kJ mol^−1^). T is temperature in Kelvin (K), R is the universal gas constant (0.0083144 kJ mol LK^−1^).

## Results and discussion

### P-XRD characterization

**Figure 1:**
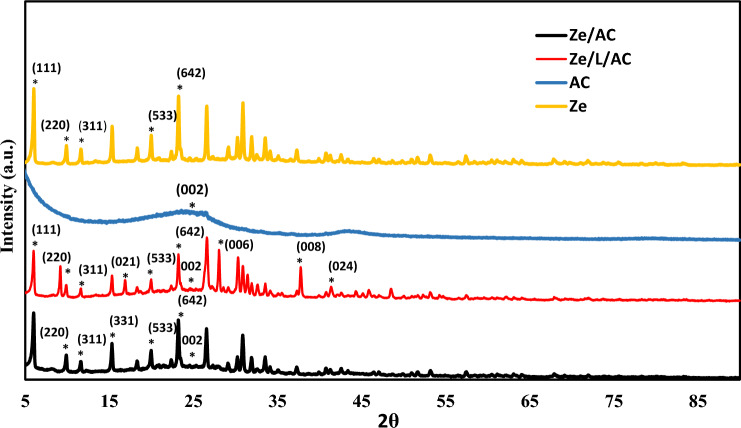
P-XRD of AC, Ze, Ze/AC and Ze/L/AC composite.

Figure 1 depicts the P-XRD analysis of activated carbon coupled with zeolite (Ze/AC) and Ze/AC with disodium terephthalate linker (Ze/L/AC). The P-XRD of these composites were compared with the P-XRD graph of pure AC and Ze. Both composites showed peaks at 6°, 10°, 12°, 15°, 20°, and 23° which were corresponded with the (111), (220), (311), (331), (533), and (642) crystal planes of Ze, respectively (JCPDS 43-0168). The round peak at 25° exhibited by activated carbon (AC), Ze/AC, and Ze/L/AC was attributed to the (002) plane of graphitic materials. The Ze/L/AC composite had peaks at 17°, 28°, 37°, and 41° that were attributed to the (021), (006), (008), and (024) planes of terephthalate linker, respectively (JCPDS 52-2146). These results were consistent with previous studies^43–45^.

### FTIR characterization

**Figure 2:**
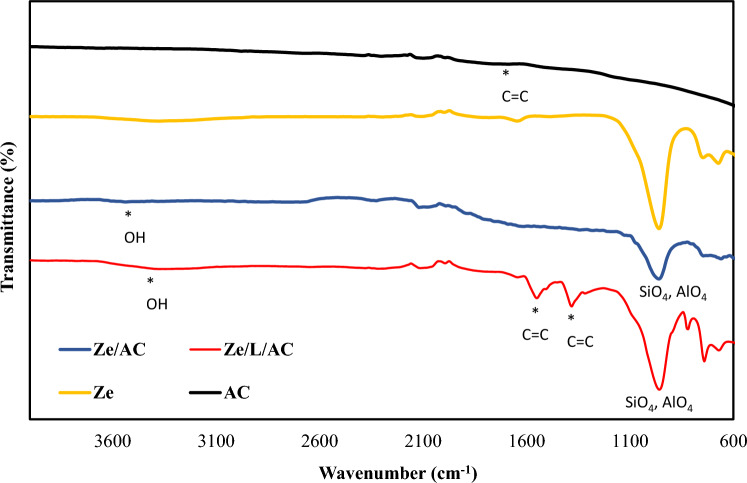
FTIR of AC, Ze, Ze/AC and Ze/L/AC.

Figure 2 shows the FTIR of Ze/AC mixture and Ze/L/AC composite compared with that of activated carbon and zeolite 13X. The band at 956 cm^−1^ and 740 cm^−1^ of Ze/AC and Ze/L/AC correspond with the asymmetrical and symmetrical stretching vibration of SiO_4_ and AlO_4_ of zeolite 13X^46^. The band at 3510–3348 cm^−1^ can be attributed to the hydroxyl group^47^. The other bands of Ze/L/AC composite at 1550 cm^−1^ and 1381 cm^−1^ indicate the C=C stretching vibration of aromatic group in disodium terephthalate^48^. The round weak peaks at 1620–1500 cm^2^ are attributed to the C=C of activated carbon^49,50^.

### SEM

**Figure 3:**
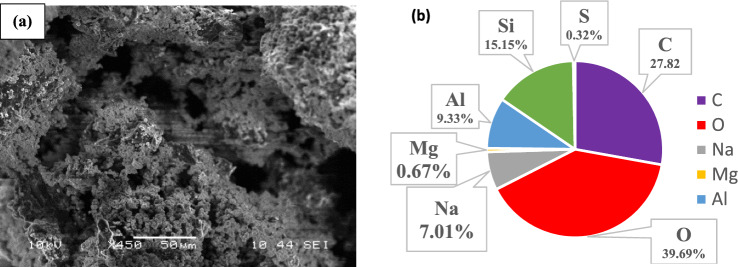
(**a**) The SEM of Ze/AC, and (**b**) The EDS of Ze/AC.

**Figure 4:**
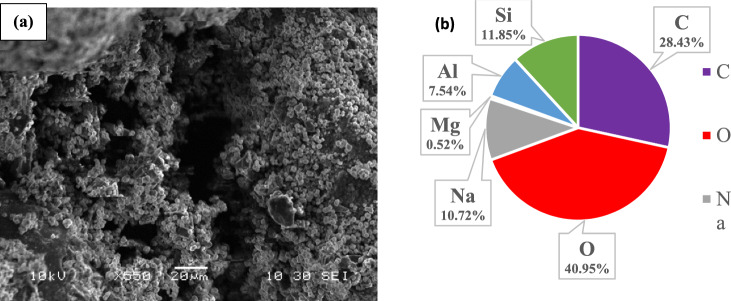
(**a**) The SEM of Ze/L/AC, and (**b**) The EDS of Ze/L/AC.

Figures 3a and 4a showed the porous surface morphology of Ze/AC mixture and Ze/L/AC composite materials, respectively. Both Figs. 3b and 4b showed the presence of dominant elements including C, Si, O, Al, and Na. The activated carbon contributed the majority of carbon weight percentage, while the weight percentages of silica, oxygen, and aluminum were from the Ze. The presence of terephthalic linker increased the weight percentages of carbon, oxygen, and sodium in the Ze/L/AC.

### TGA

The supplementary Figure S1 depicts the thermal stability of Ze/L/AC composite. The first and weight loss was seen from 16.2% from 25 to 424 °C, which was attributed to the moisture loss^50,51^. The weight loss percentages of 9.4% from 449 to 649 °C was from the degradation of the linker. Lastly, the 25% weight loss was from the decomposition of carbon^52^. The remnant 47% weight of Ze/L/AC composite was from Ze. In the study of Masika and Mokaya 2013, it found that zeolite was thermally stable up to 1000 °C^53^.

### Determination of surface area

**Figure 5:**
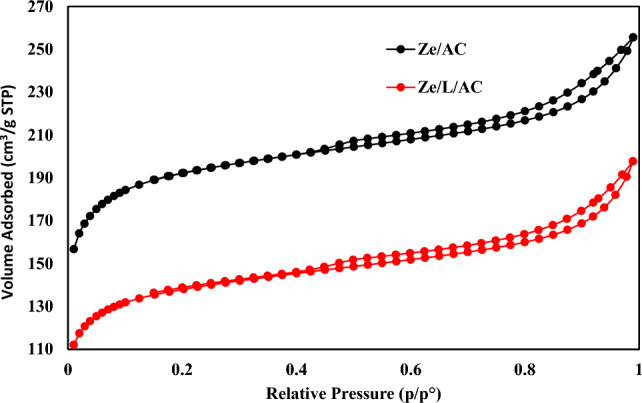
Nitrogen adsorption–desorption isotherm of Ze/AC and Ze/L/AC.

The surface areas of Ze/AC and Ze/L/AC composites were measured by a high-performance adsorption analyzer. The quantities of nitrogen adsorption and desorption are depicted in Fig. 5. Through the adsorption process, it was calculated that the BET surface areas of Ze/AC mixture and Ze/L/AC composite were 618 m^2^/g, and 445 m^2^/g, respectively. The average adsorption pore diameters of Ze/AC and Ze/L/AC composite were 2.56 nm and 2.74 nm. The large pore size for Ze/L/AC composite may improve the adsorption kinetic compared to Ze/AC.

### Adsorbent study

**Figure 6:**
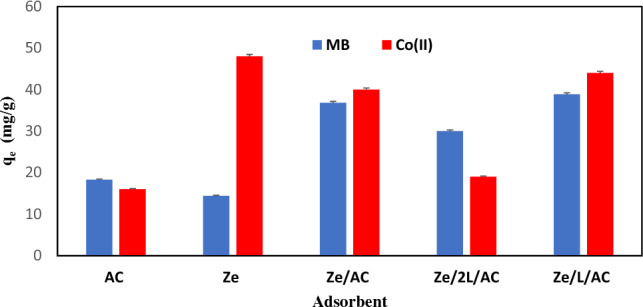
The screening MB adsorption capacity and Co(II) adsorption capacity of AC, Ze, Ze/AC, Ze/2L/AC, and Ze/LAC.

The MB and Co(II) adsorption capacities of various adsorbents (AC, Ze, Ze/AC, Ze/2L/AC, Ze/LAC) were depicted in Fig. 6. The adsorption capacities were calculated by using Eq. (1). The composite Ze/L/AC was found to have absorbed the highest amount of methylene blue with each gram of Ze/L/AC capable of adsorbing roughly 39 mg of MB. Ze adsorbed the lowest amount of methylene blue (14 mg per gram) but appeared to be highly effective in adsorbing Co (II) (48 mg per gram). When Ze was mechanically coupled with AC or linker, the MB adsorption capacity of these composites were significantly increased over their individual components. The amount of adsorbed MB per gram of Ze/AC, Ze/2L/AC, and Ze/L/AC was found to be higher than that on Ze and AC When measuring Co (II) adsorption of Ze/AC, Ze/2L/AC, and Ze/LAC, all composites adsorbed slightly less than Ze, but much higher than AC. When testing the suitable synthesis ratio for making zeolite coupled with linker and activated carbon, it was observed that the composite Ze/2L/AC had less adsorption capacities than the Ze/L/AC. The screening results demonstrated that Ze/Ac and Ze/L/AC composites offer a great promise for removing environmental pollutants.

### pH study

**Figure 7:**
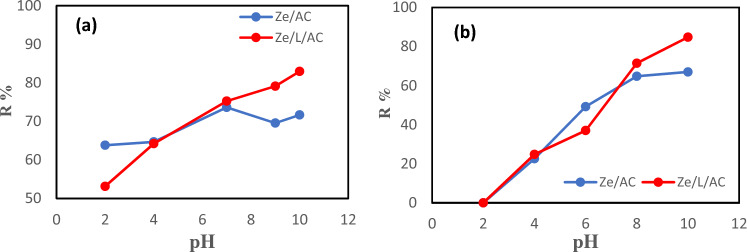
Effects of pH on (**a**) MB removal by Ze/AC and Ze/L/AC composite, (**b**) Co(II) removal by Ze/AC and Ze/L/AC composites.

Figure 7 shows the percentage removal of methylene blue and Co(II) ions for Ze/AC and Ze/L/AC. The removal percentages were calculated by using Eq. (2). In general, it has been observed that as the pH value of a solution increases, so does the percent removal^46,47^. In our MB adsorption pH study, Ze/AC achieved the highest adsorption of 75% at pH 7, while the Ze/L/AC showed the highest adsorption of 83% at pH 10. At pH’s above 7, Co(II) ions are precipitated as Co(OH)_2_^40^. Therefore, the high percentage of Co(II) removal at pH 8 and 10 are due to both precipitation and adsorption process. Similar MB and Co(II) adsorption trends have been reported in other studies^54,55^.

### Ion competition study

**Figure 8:**
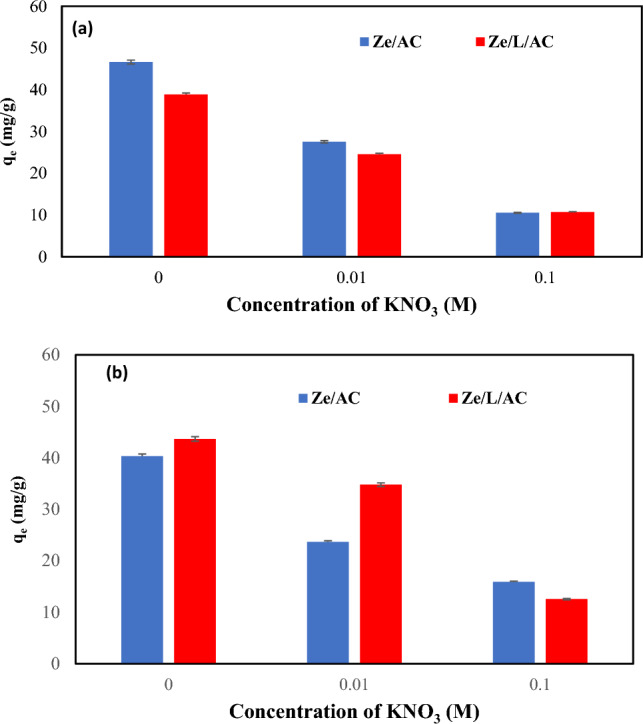
The effect of various concentration of KNO_3_ (0 M, 0.01 M, 0.1 M) on the (**a**) MB adsorption capacities of Ze/AC and Ze/LAC, (**b**) Co(II) adsorption capacities of Ze/AC and Ze/L/AC.

The ionic strength study was shown in Fig. 8. In aqueous media, KNO_3_ disassociated into potassium ions (K^+^) and nitrates ions (NO_3_^-^) which competed with Co(II) ions and MB for the surface of Ze/AC and Ze/L/AC.

Therefore, the stronger the ionic strength of a species is the stronger its adsorptive ability. It was found that both Ze/AC and Ze/L/AC performed much better in the absence of potassium nitrate (KNO_3_) with adsorption capacities of MB of 47 mg/g and 39 mg/g, respectively. The composites were found to preform similarly in the Co(II) adsorption studies with adsorption capacities (40 mg/g) and (44 mg/g) for Ze/AC and Ze/L/AC, respectively.

### Temperature study

**Figure 9:**
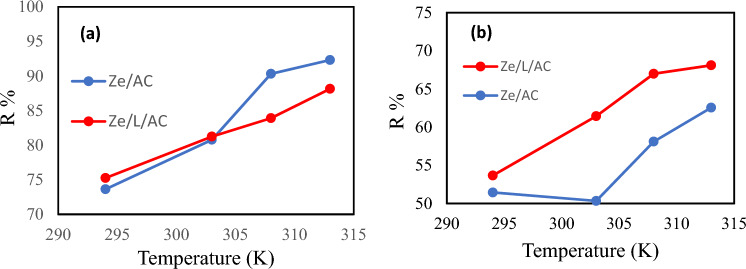
The effect of temperatures (294 K, 303 K, 308 K, 313 K) on (**a**) the percentage of MB removed by Ze/AC and Ze/L/AC, and (**b**) the percentage of Co(II) removed by Ze/AC and Ze/L/AC.

The adsorption of MB and Co(II) was conducted at 294 K, 303 K, 308 K, 313 K as depicted in Fig. 9a and 9b. As the solution temperature was increased, the performance of each adsorbent also improved. In the MB adsorption process, the percentage of MB adsorbed by Ze/AC at 308 K (90%) and 313 K (92%) were higher than those of Ze/L/AC. However, in the Co(II) adsorption, Ze/L/AC outperformed Ze/AC at all temperatures. It highly indicated that Ze/L/AC was more suitable in heavy metal adsorption.

The van’t Hoff plots depicted in the supplementary Figure S2 and Figure S3 show negative slopes which indicates that the adsorption processes are endothermic. Table 1 presents the thermodynamic parameters ΔG, ΔH, and ΔS calculated using Eqs. (9) and (10). All ΔG values were found to be negative which indicates that all the reactions were spontaneous. ΔH and ΔS of the Co (II) adsorption processes were lower than those of MB adsorption processes. These results indicated that the materials were more energetically favorable in heavy metal adsorption.

**Table 1:** Thermodynamic parameters for Ze/AC and Ze/L/AC composites in the adsorption of MB and Co(II) at 294 K, 303 K, 308 K, 313 K.

Adsorbent	Adsorbate	∆G (kJ mol^−1^)				∆H (kJ mol^−1^)	∆S (kJ mol^−1^.K^−1^)	R^2^
		294 K	303 K	308 K	313 K			
Ze/AC	MB	− 2.56	− 3.65	− 5.63	− 6.36	59.13	0.21	0.94
Ze/L/AC	MB	− 2.68	− 3.65	− 4.25	− 5.18	34.9	0.13	0.98
Ze/AC	Co	− 0.14	− 0.03	− 0.84	− 1.34	18.9	0.06	0.71
Ze/L/AC	Co	− 0.36	− 1.17	− 1.81	− 1.97	26.0	0.09	0.97

### Kinetic study

The adsorption capacities over time of Ze/AC and Ze/L/AC were calculated by Eq. (1) and shown in supplementary Figure S4 and Figure S5. It was observed that the adsorption capacity of each composite increased with time. In the MB adsorptions, Ze/AC achieved the highest adsorption capacity (36 mg/g) after 21 h, while Ze/L/AC achieved the highest adsorption capacity after 20 h. For the Co(II) adsorptions, Ze/AC and Ze/L/AC achieved the maximum adsorption capacities in the first three hours. The adsorption data was evaluated by using a pseudo-first-kinetic model and pseudo-second kinetic-model and is presented in Table 2. The adsorption rate constants of models were calculated by using Eq. (5), (6) and (7). The correlation coefficients R^2^ of Co(II) adsorption process (0.90–0.95) was higher than that of MB adsorption process (0.81–0.94). Both first order and second order models showed that the Co(II) adsorption rate constants were higher than the MB adsorption rate constants. The Co(II) adsorption first order rate constant of Ze/L/AC (4.8 h^−1^) was higher than that of Ze/AC (0.6 h^−1^). Similarly, the second order rate constant of Ze/L/AC was also higher than those of Ze/AC. The kinetic data indicates that, Ze/L/AC absorbs contaminants faster than Ze/AC possibly due to the larger pore size of Ze/AC, as indicated in our surface area study. Based on the previous studies, the well fitted pseudo-second-order indicated that chemisorption was involved^56–66^.

**Table 2:** Pseudo-first-Order and Pseudo-second-Order Model Parameters for the Adsorption of MB and Co(II).

Adsorbent	Adsorbate	Pseudo first-order kinetic model		Pseudo second-order kinetic model	
		k_1_ (h^−1^)	R^2^	k_2_ (mg^−1^ h^−1^)	R^2^
Ze/AC	MB	0.01	0.86	0.002	0.81
Ze/L/AC	MB	0.02	0.94	0.005	0.94
Ze/AC	Co(II)	0.6	0.94	0.001	0.95
Ze/L/AC	Co(II)	4.8	0.86	0.006	0.90

### Isotherm study

**Figure 10:**
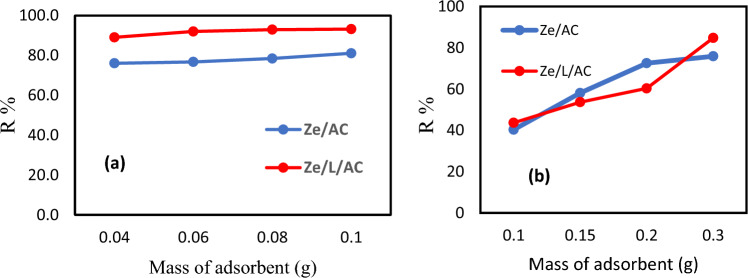
The removal percentage of (**a**) MB and (**b**) Co(II) by various masses of Ze/AC and Ze/L/AC.

The adsorption capacities of various masses of Ze/AC and Ze/L/AC on MB and Co(II) are shown in Fig. 10(a) and Fig. 10(b). For both MB and Co(II) adsorption, when the mass of adsorbents increased, the amount of adsorbed MB and Co(II) increased. Figure 10(a) indicated that the percentages of MB removed by various masses of Ze/L/AC was higher than that of Ze/AC. In Fig. 10(b), the percentages of Co(II) removed by Ze/L/AC was higher than that of Ze/AC at 0.1 g and 0.3 g.

The isotherm adsorption data was evaluated by using Langmuir and Freundlich Eqs. (3) and (4). The calculated parameters based on Langmuir and Freundlich models are depicted in Table 3. The Freundlich model shows that the Ze/AC and Ze/L/AC had higher correlation coefficients (R^2^) of 0.96 and 0.97 for MB adsorption, while the Langmuir model showed the highest correlation coefficients of 0.94 and 0.92 for the Co(II) adsorption of Ze/AC and Ze/L/AC, respectively. These results indicate that the MB adsorption data better fits the Freundlich model, while the Co(II) adsorption data produced a better fit with the Langmuir model. The Langmuir model indicates that the maximum MB adsorption capacities (Q_m_) of Ze/AC and Ze/L/AC were 67.6 mg/g and 66.6 mg/g, respectively. As shown in the Langmuir model, The Langmuir constant of Ze/L/AC (0.02) was higher than that of Ze/AC (0.003). This indicates that Ze/L/AC has a stronger affinity with Co(II) than Ze/AC. In the MB adsorption, the Ze/L/AC also demonstrated stronger interaction with MB than Ze/AC. The Freundlich constant (K_f_) of Ze/L/AC (19.01) was much higher than that of Ze/AC (0.05).

**Table 3:** Langmuir and Freundlich model’s parameters and the corresponding correlation coefficients.

Adsorbent	Adsorbate	Langmuir model parameters			Freundlich model parameters		
		K_L_	Q_m_ (mg/g)	R^2^	K_f_	n	R^2^
Ze/AC	MB	0.26	67.6	0.95	0.05	0.15	0.96
Ze/L/AC	MB	0.003	66.6	0.93	19.01	1.25	0.97
Ze/AC	Co(II)	0.003	66.7	0.94	1.53	1.9	0.90
Ze/L/AC	Co(II)	0.02	40.8	0.92	7.06	3.7	0.64

Tables 4 and 5 list the Q_m_ of this studies and other published studies. Both Tables 4 and 5 show that the saturated adsorption capacities of Ze/AC and Ze/L/AC for Co(II) and MB were higher than other reported materials. These results indicate that Ze/AC and Ze/L/AC were equally effective in removing Co(II) and MB. The mechanical synthesis method appears to have greatly improved the heavy metal and methylene blue adsorption capabilities of the composites over Ze and AC.

**Table 4:** Comparison of adsorption capacity of Co(II) onto different adsorbents.

Adsorbent for Co(II)	Q_m_ (mg/g)	References
Zeolite 13X	4	^67^
Hydroxyapatite/zeolite / composite	11	^68^
Na-Y Zeolite	54.1	^69^
Natural zeolite	0.011	^70^
Zeolite synthesized from coal gangue	18	^71^
Jordan Low- Cost Zeolite	2.73	^72^
Molecular sieves / (zeolite)	8.2	^73^
Ze/AC	66.7	This work
Ze/L/AC	40.8	This work

**Table 5:** Comparison of adsorption capacity of MB onto different adsorbents.

Adsorbent for MB	Q_m_ (mg/g)	References
clay-biochar composites	7.90	^74^
Natural Zeolite	29.18	^75^
Chitosan modified zeolite	37.04	^76^
Activated lignin-chitosan extruded	36.25	^77^
Orange Peels biomass	14.16	^78^
Zeolite-X	1.93	^79^
PDA-rGO-kaolin	39.663	^80^
Ze/AC	67.6	This work
Ze/L/AC	66.6	This work

### Adsorbate, adsorbent and proposed adsorption mechanism

**Figure 11:**
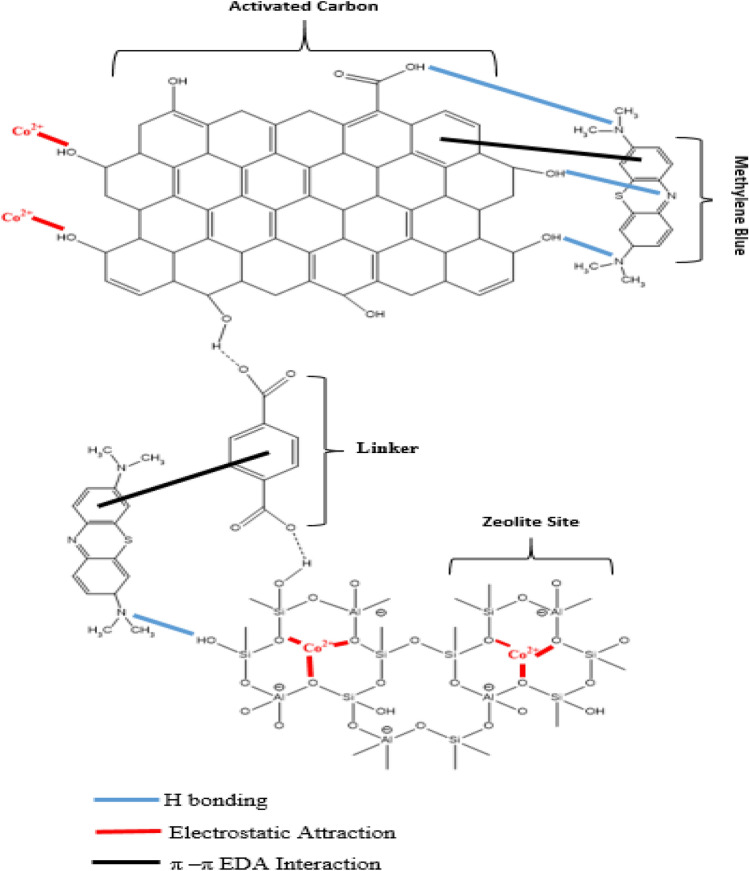
Proposed adsorption mechanism of Co(II) and methylene blue (MB) by Ze/L/AC.

Mechanochemistry refers to the use of mechanical force, such as grinding to drive chemical reactions^36–38^. The mechanical energy input promotes bond breakage, formation, or rearrangement, leading to chemical transformations^36–38^. This activation of bonds makes them more susceptible to undergoing condensation chemical reaction to remove water molecules via the intense mechanical energy generated during grinding can lead to localized heating of the reactant particles. This heating can increase the condensation rate by providing the necessary activation energy for bond breaking and formation. In addition, the high surface area generated by the grinding process increases the chances of molecular collisions between reactant particles. As a result, solid reactant particles undergo rearrangement and redistribution of atoms during the grinding process, leading to the formation of new products^36–38^. The mechanical force applied during grinding disrupted the hydroxyl group (-OH) function groups on the surface of zeolites and activated carbon in the reactant linker function group –COOH as shown in Fig. 11 as dashed lines.

Also, Fig. 11 shows the proposed adsorption sites for Ze/L/AC composite which are capable of adsorbing Co(II) via ion exchange process or electrostatic attraction. The Fan et al. 2021 study concluded that heavy metals with high electronegativity displayed a strong affinity to the negatively charged O in Si–O–Al structure^81^. Similarly, the OH groups of activated carbon can electrostatically bond with Co(II) ions^82^. Furthermore, both zeolite X and activated carbon (AC) can bind the nitrogen atoms of methylene blue through hydrogen bonding. In the Ahmad et al. 2012 study, the amino group of MB forms hydrogen bonds with the activated carbon surface which resulted from the localization of the charge^83^. The activated carbon was also able to adsorb an organic substance like methylene blue through electron donor–acceptor (EDA) interaction (π-π EDA interaction). The π-π EDA interaction mechanism was explained in Dimitrios et al. and Wheeler studies^83,84^. The π-π EDA interaction are optimized when one ring is substituted with electron donors and the other with electron withdrawing groups [85]. The linker is also capable of assisting the composite in adsorbing MB through the π-π EDA interaction.

## Conclusion

The study successfully synthesized new Zeolite with activated carbon (Ze/AC and Ze/L/AC) composites via liquid-assisted grinding (LAG). Both composites showed a high efficiency in removing Co(II) and MB from aqueous media. The Ze/AC and Ze/L/AC had higher maximum adsorption capacities than other similar reported materials. The capacities of Ze/AC in removing MB and Co(II) from aqueous media was 67.6 mg/g and 66.7 mg/g, while the MB and Co(II) adsorption capacities of Ze/L/AC was 66.6 mg/g and 44.8 mg/g. However, Ze/L/AC (4.8 h^−1^) has faster absorption rate than Ze/AC (0.6 h^−1^). This study offers insight into the extraordinary potential for applying LAG mechano-synthesis for creating effective adsorbents for various environmental applications in the future. The multifunctional composite is not only able to remove metal ions but also organic contaminants.

### Supplementary Information

Supplementary Information.

## Data Availability

The data presented in this study are available on request from the corresponding author.
