# Supplementary material for: Synthesis of a novel multifunctional organic–inorganic nanocomposite for metal ions and organic dye removals
Source: Sci Rep. 2023 Aug 8;13:12845. doi: 10.1038/s41598-023-38420-2 (PMC10409728; doi:10.1038/s41598-023-38420-2)
Supplement: Supplementary file 1 — Supplementary Information. [file 41598_2023_38420_MOESM1_ESM.docx]

**Supplementary Material**

**Synthesis of a Novel** **Multifunctional Organic-Inorganic Nanocomposite for Metal Ions and Organic Dye Removals**

Ahmed Elmekawy^1,2^, Qui Quach,^1^ and Tarek M. Abdel-Fattah^1,3^*

^1^Applied Research Center at Thomas Jefferson National Accelerator Facility and Department of Molecular Biology and Chemistry at Christopher Newport University, Newport News, VA 23606, USA

^2^Department of Physics at Tanta University, Tanta, Al Gharbiyah, Egypt

^3^Faculty of Sciences, Alexandria University, P.O. Box 426, Ibrahimia, 21321 Alexandria, Egypt

**Corresponding Author email: fattah@cnu.edu*

**Figure S1:** TGA graph of Ze/L/AC

**Figure S2**. The van’t Hoff plot for the MB adsorption of Ze/AC and Ze/L/AC at the temperatures of 294 K, 303 K, 308 K, and 313K

**Figure S3**: The van’t Hoff plot for the Co(II) adsorption of Ze/AC and Ze/L/AC at the temperatures of 294 K, 303 K, 308 K, and 313K

**Figure S4**: The MB adsorption capacities (mg/g) of Ze/AC and Ze/L/AC for 24 hours.

**Figure S5**: The Co(II) adsorption capacities (mg/g) of Ze/AC and Ze/L/AC for 24 hours.
